# Effect of Pre-Rolling on Microstructure and Fatigue Crack Propagation Resistance of a Third-Generation Al-Li Alloy

**DOI:** 10.3390/ma16247540

**Published:** 2023-12-07

**Authors:** Meng Liu, Xiaoyu Tao, Zhiyu Di, Mengli Qin, Zhiyi Liu, Song Bai

**Affiliations:** 1 School of Materials and Chemical Engineering, Pingxiang University, Pingxiang 337055, China; 2 School of Material Science and Engineering, Central South University, Changsha 410083, China

**Keywords:** Al-Cu-Li alloy, fatigue crack propagation, T_1_ phase, pre-rolling, microstructure

## Abstract

The effect of pre-rolling on the microstructure and fatigue crack (FC) propagation resistance of the Al-Cu-Li alloy was studied using tensile testing, fatigue testing, transmission electron microscopy (TEM), X-ray diffractometer (XRD), and scanning electron microscopy (SEM). The results revealed that reducing the alloy thickness through pre-rolling by up to 12% enhanced both tensile strength and yield strength, albeit at the expense of reduced elongation. In addition, the FC growth rate decreased by up to 9% pre-rolling, reaching the minimum, while the application of additional mechanical stress during the pre-rolling increases this parameter. Deformations in the Al-Cu-Li alloy with less than a 9% thickness reduction were confined to the surface layer and did not extend to the central layer. This non-uniform deformation induced a compressive stress gradient in the thickness direction and led to an inhomogeneous distribution of T_1_ phase, resembling the structure generated by shot peening. The superior FC propagation resistance in the 9% pre-rolled alloy could be primarily attributed to the optimum balance of compressive residual stress and work hardening.

## 1. Introduction

Third-generation Al-Li alloys have found extensive use in various aircraft structural components owing to their unique blend of qualities, including low density, high strength, specific excellent stiffness, and exceptional toughness [1,2,3]. In the context of aerospace applications, where Al-Li alloys are predominantly employed, their performance under cyclic loading conditions becomes paramount. Thus, fatigue performance is a critical factor that can either facilitate or restrict the broader application of Al-Li alloys. 

The resistance to fatigue crack (FC) propagation in Al-based alloys is a complex function of their microstructure. Key factors include the type, size, number, and distribution of precipitates [4], as well as grain size and orientation [5], and the distribution of residual stress [6]. In the case of age-hardenable Al alloys, precipitates are a significant parameter in shaping the fatigue properties of Al-Li alloys [7,8,9]. Compared to the second generation of Al-Li alloys, the third generation typically contains a lower Li content, ranging from 1 to 1.8 wt.%. This reduction in Li content inhibits the formation of the δ′ (Al_3_Li) phase and promotes the T_1_(Al_2_CuLi) phase as the primary strengthening agent [10]. 

The hexagonal T_1_ phase takes shape as semi-coherent platelets along the {111}_Al_ plane of the base material and has a preference for precipitating at (sub-) grain interfaces and along dislocations [11]. On the other hand, the coherent δ′ phase is prone to shearing by dislocations, causing them to move along the existing slip plane, leading to planar slip. This planar slip phenomenon has been observed to decrease fatigue resistance due to inhomogeneous plastic deformations in the alloy [12]. In contrast, the third-generation Al-Li alloy introduces T_1_ precipitates that are resistant to shearing by dislocations, resulting in a shift from planar slip to wave slip, thereby enhancing the deformation uniformity within the microstructure [13]. For example, Zhang et al. [14] investigated the impact of precipitation on the fatigue properties of the Al-Li alloy and observed that the existence of the T_1_ phase increases the uniformity of plastic deformations under cyclic loading, thus improving the fatigue lifetime of the alloy. Rao et al. [15] reported that Al-Li alloys display significant crack shielding, attributed to the deflection of crack path, thus impeding the propagation of long cracks. Cisko et al. [16] proposed that Al-Li alloys exhibit enhanced fatigue resistance associated with the T_1_ phase, which can lead to crack deflection and impede the propagation of FCs. Therefore, the rational control of precipitates represents an effective strategy for improving the fatigue resistance of Al-Li alloys. 

The distribution of residual stress also influences the fatigue properties of the alloy. Previous studies have shown that the introduction of compressive residual stress can effectively enhance the fatigue strength and fatigue life of the alloy [6,17]. In industrial applications, shot peening is a widely adopted mechanical surface treatment known to improve the fatigue resistance of metallic components. This process imparts specific residual compressive stresses to the material’s surface, thereby inhibiting crack initiation and extending fatigue life [18].

Chen et al. [19] investigated the impact of shot peening on the fatigue performance of a Ti alloy. Their results demonstrated a significant improvement in fatigue properties, with the high-cycle fatigue life extending by over 25 times and the fatigue endurance limit increasing by nearly 2 times. Martin et al. [20] explored the effect of shot peening on the fatigue properties of a 7075-T651 Al alloy. They found that shot peening delayed the onset of fatigue cracks by introducing high compressive residual stresses near the surface, although it also resulted in microstructural changes and alterations in surface roughness. Li et al. [21] conducted research on the effects of micro-shot peening (MSP) and conventional shot peening (CSP) on the fatigue properties of EA4T axle steel. The results indicated that, in comparison to CSP, MSP improved the compressive residual stress and reduced the roughness of the surface, thus improving the fatigue limit. In summary, the compressive residual stress is advantageous for boosting the fatigue resistance of shot-peened alloys, whereas surface roughness has a detrimental effect.

Pre-deformation is a widely utilized technique for post-quenching treatment of Al alloys, serving to reduce the residual stress generated during the quenching process and to straighten the sheets. Common pre-deformation methods include pre-stretching and pre-rolling processes. Pre-stretching significantly enhances the yield and tensile strength but diminishes fatigue crack growth (FCG) resistance due to reduced plastic zone size and a decreased crack closure effect [22,23]. Nevertheless, there exist limited data on the impact of pre-rolling on the fatigue damage tolerance of Al alloys. As noted by Zhao [24], rolling an alloy plate can yield a structure similar to the “hard surface and tenacious core” generated by shot peening. Chen et al. [25] studied the impact of pre-rolling on the FCG rate of 2195 Al-Li alloy. Their findings revealed that as the pre-rolling reduction increased, so did the density of the T_1_ phase and the yield strength of the alloy, ultimately leading to an increase in the FCG rate and a consequent reduction in fatigue life. Huang et al. [26] proposed that the damage tolerance of Al-Cu-Mg alloy sheets could be significantly enhanced with less than a 10% thickness reduction by pre-rolling. This occurs because the deformation associated with modest pre-rolling is confined to the surface layer of the sheet, sparing the central parts of the alloy, resulting in a gradual decrease in compressive residual stress from the surface to the center. 

However, the effects of microstructure heterogeneity and residual compressive stress arising from pre-rolling on the fatigue properties of Al-Cu-Li alloy remain largely unexplored. Hence, this study aims to find the relationship between microstructure and FCG rate in pre-rolled Al-Cu-Li alloys, with the goal of optimizing pre-rolling parameters to enhance their FC resistance.

## 2. Experimental Section

The material used in this study was a commercial Al-Cu-Li homogenization treatment alloy plate (Zhengzhou Light Alloy Institute Co., Ltd., Zhengzhou, China) with a thickness of 6.3 mm. This alloy comprises 3.62% Cu, 1.40% Li, 0.49% Mg, 0.49% Zn, 0.26% Mn, and 0.12 Zr% (in wt.%), with the remaining being Al. The specimens were subjected to a series of treatments, beginning with the solution treatment at 515 °C for 1.5 h, followed by rapid quenching in cold water. Subsequently, pre-rolling was performed to reduce the thickness by 0%, 3%, 6%, 9%, and 12%, and aging was carried out at 165 °C for 24 h. 

For tensile testing, specimens (30 mm in length) were prepared vertically to the longitudinal direction of the sheets. These tests were conducted on a CSS-44100 device (Changchun Test Machine Research Institute Co., Ltd., Changchun, China) operating at ambient temperature with a loading speed of 2 mm/min. All mechanical properties reported here represent the average of three independent specimens analyzed under the same conditions. Compact tension (CT) specimens for fatigue testing were prepared from the pre-deformed plates in the L-T orientation, with dimensions of 47.5 mm × 38 mm × 5.5–6.3 mm (Length × width × Thickness). Fatigue crack propagation (FCP) tests were carried out on an MTS-810 test machine (MTS Company, Eden Prairie, MN, USA) with a constant K value of K_max_ = 10 MPa·m^0.5^, a maximum load of P = 1 kN, a stress ratio (*R* = σ_min_/σ_max_) of 0.1, and a loading frequency (*f*) of 10 Hz at ambient temperature and air atmosphere. The fatigue fracture surfaces were examined using a SEM (Quanta 200, FEI Company, Hillsborough, OR, USA) operating at 20 kV. TEM analysis was performed using a Tecnai G^2^ 20 device (FEI Company, Hillsborough, OR, USA) operating at 200 kV. To prepare samples for TEM analysis, thin discs with a diameter of 3 mm were electropolished using a twin-jet device (Beijing Yulon Motor Times Technology Co., Ltd., Beijing, Chin), employing a mixture of 70% ethanol and 30% nitric acid (Pingxiang Keyang Chemical Co., Ltd., Pingxiang, China) at approximately −25 °C. Residual stresses in the thickness direction were measured using an XRD spectrometer (Rigaku D/Max 2500 PC, Rigaku Corporation, Tokyo, Japan) equipped with a Cu Kα (*λ* = 0.154 nm) source and employing a 2θ-sin^2^ψ method [27]. The XRD peaks corresponded to the (311) plane for the Al-Cu-Li alloy.

## 3. Results

### 3.1. Tensile Properties

Table 1 presents the mechanical properties of Al-Cu-Li alloys at room temperature under various pre-rolling treatments. Notably, the alloy with no pre-rolling exhibited the highest elongation but the lowest tensile strength (TS) and yield strength (YS). As the pre-rolling degree increased from 0 to 12%, the TS of the alloys increased from 475 MPa to 546 MPa, and the YS increased from 393 MPa to 525 MPa, marking a remarkable 14.9% and 33.6% improvement, respectively. However, the elongation of the alloys exhibited a gradual decline from 15.1% to 6.5% as the pre-rolling degree increased from 0 to 12%.

### 3.2. Fatigue Properties

**Figure 1:**
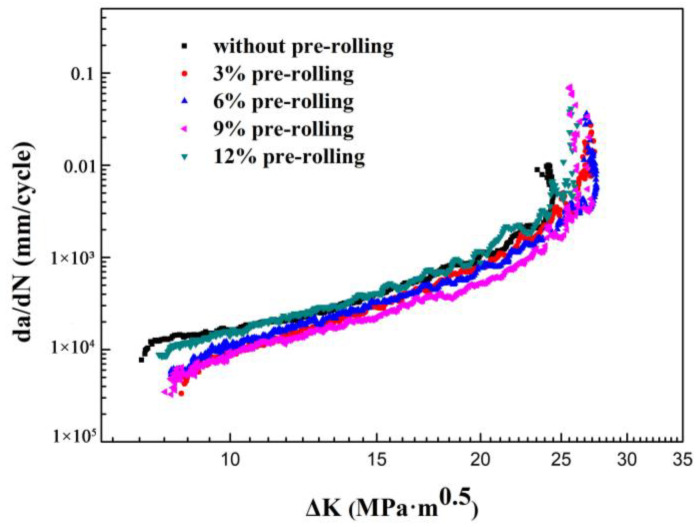
Fatigue crack propagation rates, da/dN as a function of the stress intensity factor range (∆K) for Al-Cu-Li alloy in various pre-rolling conditions.

Figure 1 demonstrates the variation in FCG rates concerning the stress intensity factor range (ΔK) for different pre-rolling conditions of the Al-Cu-Li alloy. Notably, the non-deformed sample exhibited the highest FCG rates in the near-threshold regime, with the lowest maximal ΔK values (referred to as ΔK_max_) at which fatigue fracture occurred compared to other pre-rolled Al-Cu-Li alloys. This observation indicates that pre-rolling can enhance the fatigue damage resistance of the Al-Cu-Li alloy. Furthermore, the alloys displayed varying FCG rates under different pre-rolling setups in the Paris regime. With an identical ΔK, the FCG rate initially decreased as the degree of pre-rolling increased within the range of 3% to 12%, reaching its lowest point at a 9% pre-rolled condition before subsequently increasing.

In the Paris regime of the fatigue, the Paris formula, *da*/*dN* = C(∆K)^n^, can be used to calculate the crack growth rate of the alloy. C and n are material parameters. The Paris models of the samples under the five investigated pre-rolling conditions are given as follows:

$$\frac{da}{dN}=\left\{ \begin{array}{l} 2.65\times 10^{7}{\left(\Delta K\right)}^{2.72},\;\mathrm{non-predeformed}\\ 5.96\times 10^{8}{\left(\Delta K\right)}^{3.16},\;3\%\;\mathrm{pre-rolling}\\ 2.23\times 10^{7}{\left(\Delta K\right)}^{2.69},\;6\%\;\mathrm{pre-rolling}\\ 2.58\times 10^{7}{\left(\Delta K\right)}^{2.75},\;9\%\;\mathrm{pre-rolling}\\ 2.94\times 10^{7}{\left(\Delta K\right)}^{2.48},\;12\%\;\mathrm{pre-rolling} \end{array}\right..$$

Because the values of C and n do not change obviously with pre-deformation, the application of the traditional Paris’ law to specimens with different pre-deformation is difficult.

**Figure 2:**
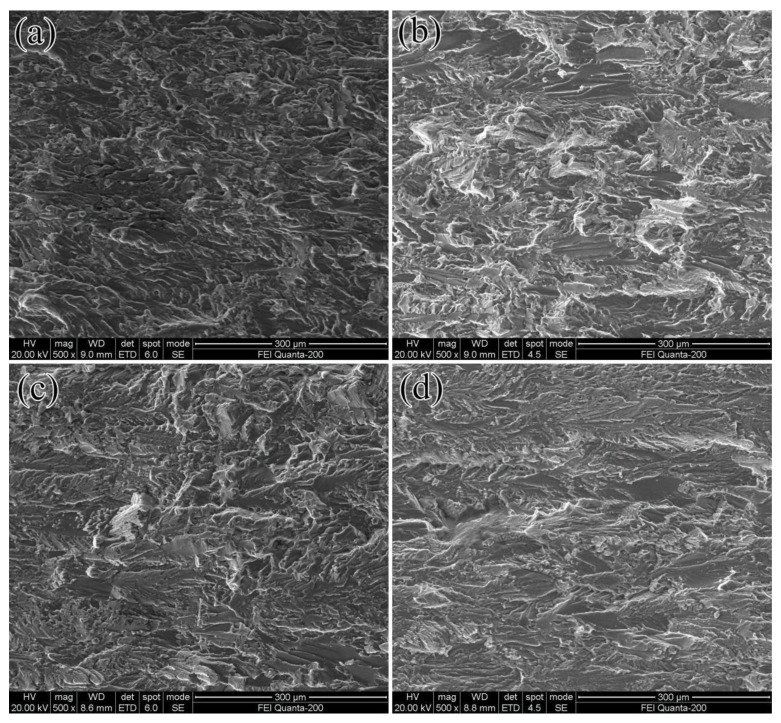
SEM (scanning electron microscopy) fractographs characterizing the fatigue fractures surfaces of various pre-deformation conditions of the Al-Cu-Li alloy in a near-threshold regime at the same ∆K of 9 MPa·m^0.5^. The fatigue crack propagated from left to right: (**a**) without pre-rolling; (**b**) 6% pre-rolling; (**c**) 9% pre-rolling; (**d**) 12% pre-rolling.

The fatigue fracture surfaces of different pre-deformation conditions of the Al-Cu-Li alloy in the near-threshold regime are shown in Figure 2. No significant differences were observed in the morphology of all samples in the near-threshold regime. The fatigue fracture morphology exhibited distinct river-like characteristics, and these river patterns roughly followed the propagation direction of the main fatigue crack from left to right. Additionally, tear ridges and several microscopic voids were observed in all fracture morphologies. These microscopic voids primarily resulted from dislocation bunching around the coarse second-phase particles in the alloy during fatigue, leading to the detachment of these second-phase particles from the matrix interface and the formation of microholes.

**Figure 3:**
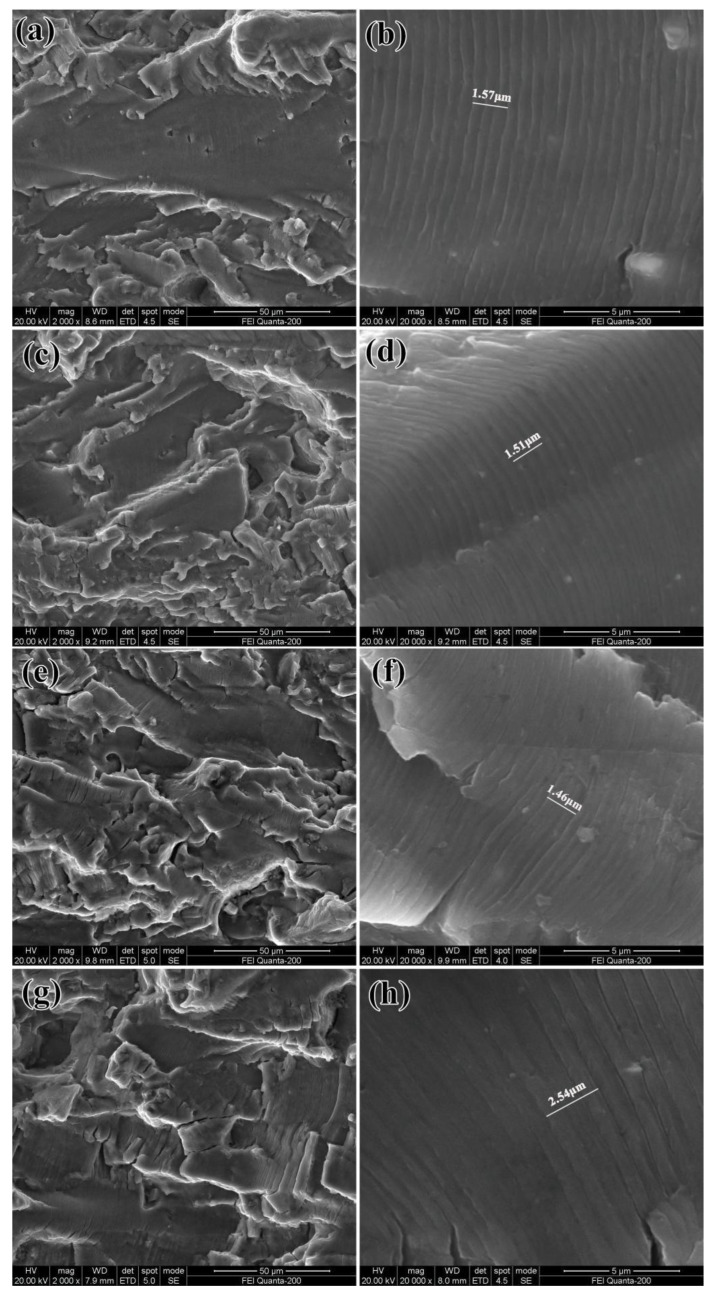
SEM fractographs characterizing the fatigue fractures surfaces of various pre-deformation conditions of Al-Cu-Li alloy in Paris region at the same ∆K of 20 MPa·m^0.5^. The fatigue crack propagated from left to right: (**a**,**b**) without pre-rolling; (**c**,**d**) 6% pre-rolling; (**e**,**f**) 9% pre-rolling; (**g**,**h**) 12% pre-rolling.

Figure 3 displays the fatigue fracture surfaces of Al-Cu-Li alloy samples prepared under various pre-deformation conditions within the Paris regime, all at the same ∆K value of 20 MPa·m^0.5^. In the low-magnification SEM images, it is evident that there are no significant variations in the morphology of the four fatigue fractures. All exhibit the characteristics of transgranular fracture, with fatigue cracks propagating from left to right. These fracture surfaces display numerous smooth crystallographic planes of varying sizes, most of which are interconnected by tearing ridges. 

Figure 3b,d,f,h provide a high-magnification view of the crystallographic planes, revealing that the fracture morphologies in the Paris region of alloys subjected to different pre-rolling conditions are strikingly similar. Each of them consists of well-defined fatigue striations, characterized by a slight curvature and parallel alignment, nearly perpendicular to the primary crack’s path. It is widely accepted that the spacing between two fatigue striations depict the forward distance of fatigue crack propagation within one stress cycle. A larger gap between fatigue striations corresponds to a higher FCG rate, signifying lower FCG resistance. As shown in Figure 3b,d,f,h, three distinct and continuous fatigue striations were selected as the measurement for fatigue striations spacing. The average of the three measurements is shown in Figure 3. The fatigue striations spacing of the four pre-rolled alloys are 1.57 μm, 1.51 μm, 1.46 μm, and 2.54 μm, respectively. FCG rate = L/D, where L is the measurement length of three fatigue striations and D is the number of striations (i.e., 3). The corresponding average FCG rates are calculated as 5.23 × 10^−4^ mm/cycle, 5.03 × 10^−4^ mm/cycle, 4.87 × 10^−4^ mm/cycle, and 8.47 × 10^−4^ mm/cycle, respectively. These findings align with the FCG rates presented in Figure 1.

**Figure 4:**
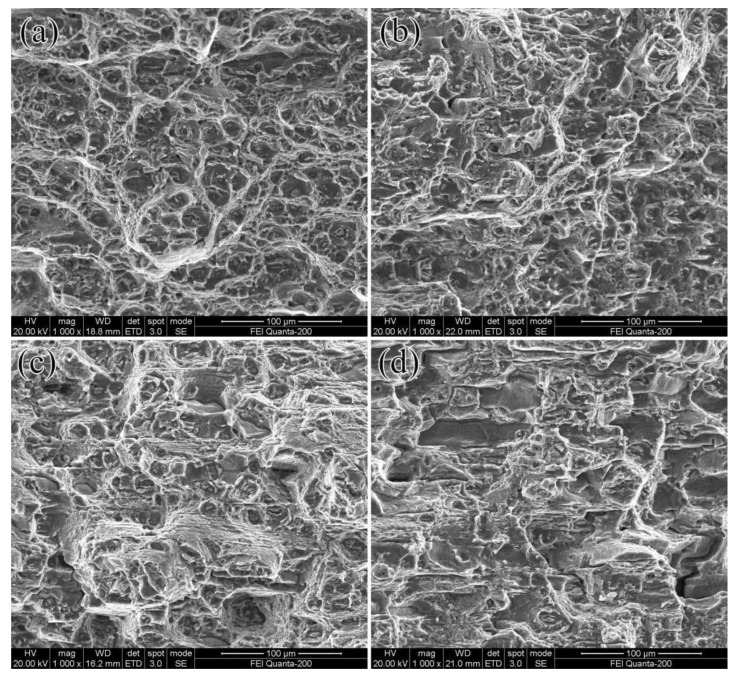
SEM fractographs characterizing the fatigue fractures surfaces of various pre-deformation conditions of Al-Cu-Li alloy in final fracture region. The fatigue crack propagated from left to right: (**a**) without pre-rolling; (**b**) 6% pre-rolling; (**c**) 9% pre-rolling; (**d**) 12% pre-rolling.

The fatigue fracture surfaces of Al-Cu-Li alloy under different pre-rolled conditions in the final fracture region are presented in Figure 4. These fractographs exhibit the typical characteristics of ductile fracture, which include clearly visible dimples [11,25]. Furthermore, the surfaces show an array of dimples and voids of various sizes, along with broken coarse second-phase particles and some noticeable tear ridges. A comparison between the pre-rolled sample and the one without pre-deformation reveals that the dimples in the latter are smaller and more uniformly distributed, indicating superior ductility. However, the 12% pre-rolled sample displayed the fewest dimples, with highly uneven dimple sizes, signifying reduced plasticity. This result is consistent with the earlier findings with respect to the tensile properties outlined in Table 1.

### 3.3. Microstructure 

**Figure 5:**
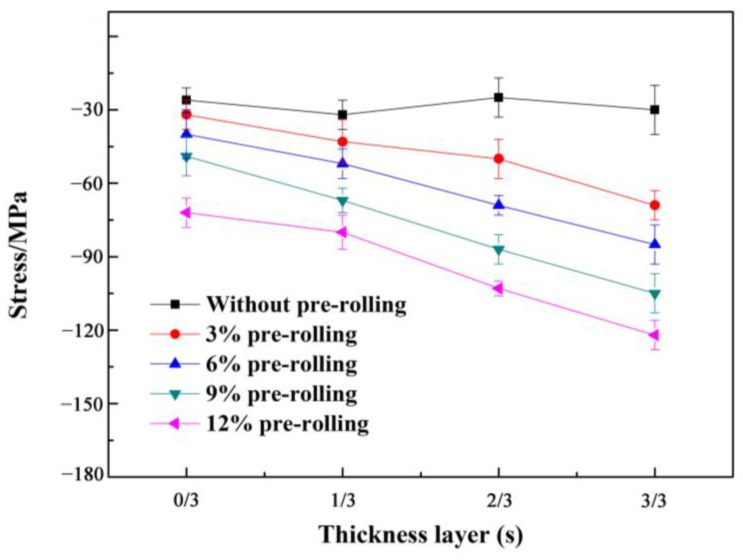
The residual stress in different thickness layers from the center layer (s = 0) to the surface layer (s = 1) of the Al-Cu-Li alloy with different pre-deformation conditions.

The impact of pre-rolling on the residual stress of alloy films is depicted in Figure 5, where negative values denote compressive stress (σ_C_), and positive values stand for tensile stress (σ_T_). In the non-deformed specimen, a small σ_C_ of about 28 MPa is uniformly distributed throughout the thickness direction. In the case of pre-rolled samples, the σ_C_ steadily increases from the central layer (s = 0) to the surface layer (s = 1) as the pre-rolling degree varies from 0 to 12%. The central σ_C_ values in 3%, 6%, and 9% pre-rolled samples were nearly equivalent to that of the non-deformed sample. However, when compared to the non-deformed sample, the central σ_C_ values in the 12% pre-rolled sample notably increased. This indicates that work-hardening progressively intensified as the pre-rolling thickness reduction increased. The higher FC growth rate observed in the 12% pre-rolled specimens in Figure 2 is a consequence of more pronounced work hardening. It is widely recognized that σ_C_ can significantly enhance the fatigue properties of metal by inhibiting FCP. Therefore, the 9% pre-rolled sample exhibited the most suitable σ_C_ gradient, contributing to its superior fatigue properties.

**Figure 6:**
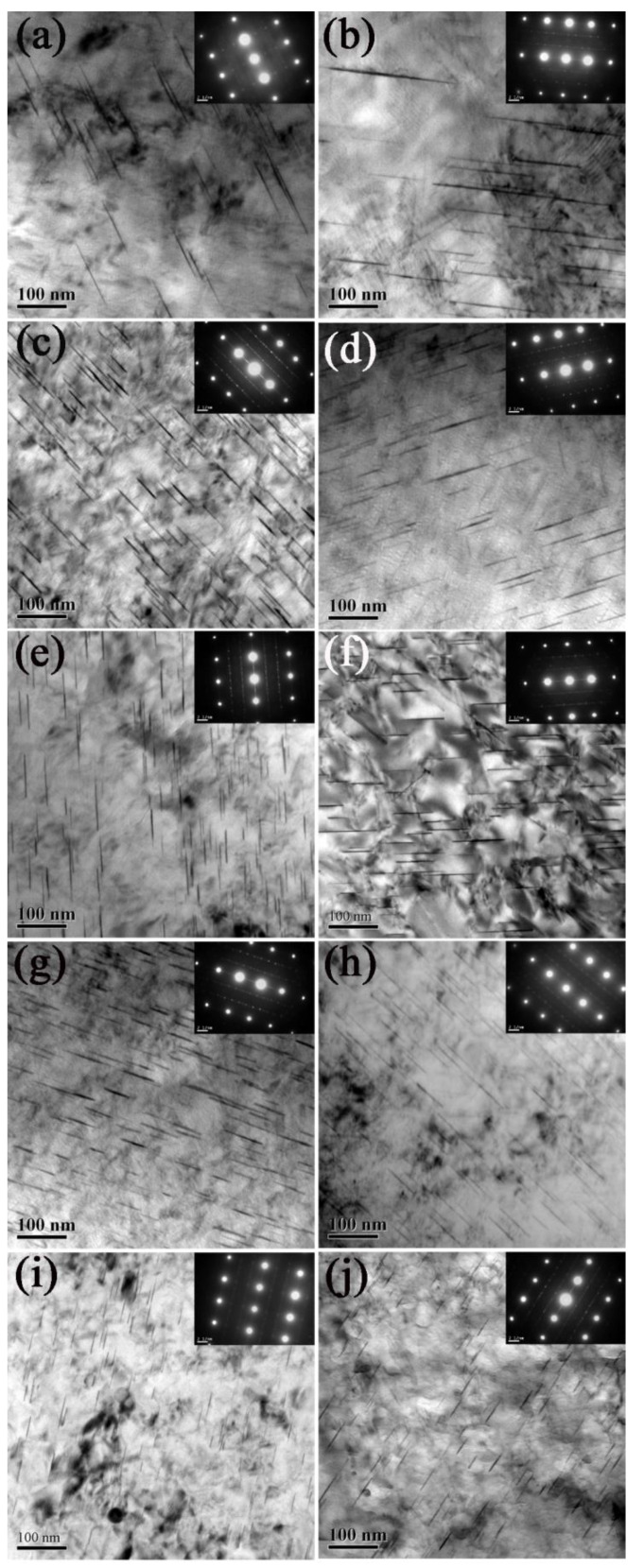
TEM images showing the typical microstructures and corresponding selected area electron diffraction (SAED) patterns of (**a**,**c**,**e**,**g**,**i**) the surface and (**b**,**d**,**f**,**h**,**j**) the center layer of Al-Cu-Li alloy with different pre-deformation conditions, the electron beam is close to <112>_α_. (**a**,**b**) Without pre-rolling; (**c**,**d**) 3% pre-rolling; (**e**,**f**) 6% pre-rolling; (**g**,**h**) 9% pre-rolling; (**i**,**j**) 12% pre-rolling.

In Figure 6, we present TEM bright field (BF) images along with the corresponding selected area electron diffraction (SAED) patterns for the surface and central layers of various pre-rolled Al-Cu-Li alloys. The electron beam was aligned close to <112>_α_ plane. The microstructure of all samples featured a white Al matrix and numerous gray, needle-like particles with a specific crystalline orientation with the Al matrix. An analysis of the corresponding SAED patterns in Figure 6 confirms that these needle-like second-phase particles correspond to the T_1_(Al_2_CuLi) phase. 

**Figure 7:**
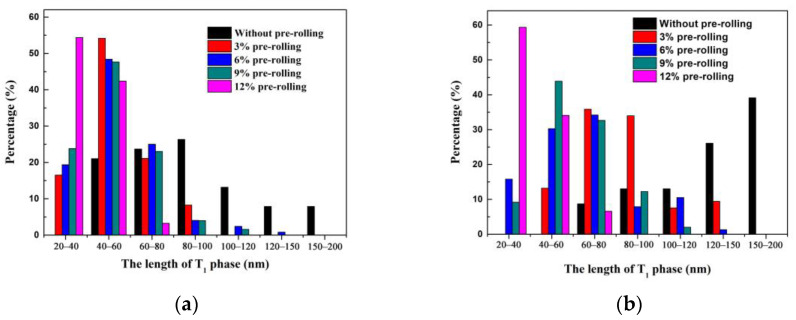
The length distribution map of T_1_ phase in various pre-deformation conditions of Al-Cu-Li alloy: (**a**) surface layers; (**b**) center layers.

To quantify the T_1_ phase, we measured the length of the T_1_ phase in TEM bright field images at the same magnification for each state sample, and the changes in the percentage of the T_1_ phase as a function of its length is shown in Figure 7. Additionally, the average length of T_1_ phase are summarized in Table 2. The distribution of T_1_ phase in the surface and central layers of the non-pre-rolled sample is more uniform, with larger-sized T_1_ phase particles (about 100 nm) and a lower quantity. In comparison with the untreated alloy, the average length of the T_1_ phase in both the central and surface layers of the pre-rolled sample is significantly reduced. As the degree of pre-rolling increases within the range of 3% to 12%, the size of the T_1_ phase particles in the surface and central layers decreases, and the quantity of T_1_ phase increases.

Specifically, in the surface layers of the 3%, 6%, and 9% pre-rolled samples, the T_1_ phase size is notably smaller than that in the central layer. However, in the case of the 12% pre-rolled samples, the size difference between the surface layer (38.87 nm) and the central layer (39.32 nm) is not significant. This is because most of the deformation in small pre-rolling (3–9%) samples occurs only in the surface layer, which is also corroborated by the residual stress results in Figure 5. In the surface layer with more dislocations, serving as nuclei for the precipitation of T_1_ phase, the number of T_1_ phases significantly increases, and their size decreases. On the other hand, large pre-rolling (12%) results in deformation throughout the entire cross-section of the plate, affecting both central and surface layers. Consequently, numerous dislocations form throughout the entire alloy, leading to the uniform dispersion of T_1_ phase.

## 4. Discussion

The strengthening effect of deformation heat treatment results from a combination of work hardening and precipitation strengthening. Pre-deformation treatment performed before aging plays a significant role in increasing the dislocation density in the alloy, leading to a notable work-hardening effect. The presence of a high density of dislocations promotes the segregation of solute atoms, providing sites for the nucleation of the T_1_ phase and accelerating the precipitation kinetics of the T_1_ phase.

It is widely recognized that precipitation strengthening constitutes the primary mechanism of enhancing the strength of Al-Cu-Li alloys, relying on dislocation-precipitate interactions [28]. The behavior of dislocations within the microstructure involves either shearing the precipitate particles or forming dislocation loops around them, depending on the size of these particles [29]. Dislocations shear particles when the precipitate radius is smaller than a critical value. The increment of yield strength Δ*σ*_s_, attributed to the shearing mechanism, is described by the following equation:

(1) $$\Delta \sigma_{s}=\beta f^{1/2}r^{1/2}.$$

Here, *β* is a constant, while *r* and *f* stand for the radius and volume fraction of the particles, respectively [29]. As this equation illustrates, Δ*σ*_s_ can be increased by enhancing the radius and volume fraction of shearable particles. In contrast, when the radius of the precipitate particles reaches the critical value, dislocations bypass them. In this scenario, the increment of yield strength Δσ_s_ is governed by the following equation:

(2) $$\Delta \sigma_{s}=\alpha f^{1/2}r^{-1}$$

In this equation, *α* represents a constant [30]. This equation demonstrates that increasing the volume fraction and decreasing the radius of the particles are beneficial to improving Δ*σ*_s_.

Figure 6 illustrates that the T_1_ phase is the main precipitate within the Al-Cu-Li alloy, making the size and density of the T_1_ phase critical factors in influencing the mechanical properties of the alloy. Studies by Jata [31] and Howe [32] have reported that dislocations are capable of shearing T_1_ phase particles. In contrast, Sainfort [33] observed that T_1_ phases could only be bypassed, not sheared, by dislocations. Additional research [34] has revealed that dislocations can shear coherent precipitates (δ′) but bypass semi-coherent precipitates (θ′, Ω, and T_1_), as well as non-coherent precipitates (S). Blankenship [35] suggested that the ability of the T_1_ phase to be sheared by dislocations depends on its size. When the phase size is smaller than the critical size, dislocation can cut through them, but when the size exceeds the critical size, dislocations can only bypass them. Blankenship [36] also proposed a method by which to determine the critical size (d_c_) of the T_1_ phase:

d_c_ = 4πbG_m_/G_p_. (3)

Here, G_m_ and G_p_ denote the shear modulus of the matrix and the particles, while b stands for the Bergdahl vector, and d_c_ represents the critical diameter. Using this equation, Li et al. [37] calculated that the critical thickness of the T_1_ phase is 0.8226 nm. By examining the TEM images of the T_1_ phase in this study (see Figure 6), it becomes evident that the average thickness of the T_1_ phase significantly exceeds the critical size. This implies that the T_1_ phase in this alloy cannot be sheared by dislocations. Referring to Equation (2), the increase in yield strength Δσ_s_ resulting from precipitation strengthening is inversely proportional to the size of the T_1_ phase. The higher yield strength in pre-rolled alloys compared to the non-pre-deformed sample is attributed not only to the work hardening induced by pre-deformation but also to the smaller size and higher density of the T_1_ phase in the microstructure, as depicted in Figure 6. Furthermore, as the extent of pre-rolling increases from 0 to 12%, the yield strength continues to rise due to the reduction in the T_1_ phase and the increase in T_1_ phase density. 

Schijve’s research [38] demonstrated that the crack growth rate in the pre-strained specimen increased with higher yield strength in comparison to the non-deformed analog. This phenomenon was attributed to the reduced size of plastic deformation zones at the crack tip in the specimens with higher yield strength. A smaller plastic zone leads to reduced crack closure and increased stress accumulation in the crack-tip region. Furthermore, pre-straining induced a substantial increase in dislocations and enhanced the interactions between these dislocations and second-phase particles. Second-phase particles are regarded as preferred sites for the initiation of micro-damage. When dislocations come into contact with these particles, their accumulation around the particles results in local stress concentration, leading to the formation of micro-cracks. Therefore, the increase in dislocation density associated with pre-straining amplifies the interaction between precipitates and dislocations, thereby increasing the mechanical stress that results in the generation of additional micro-cracks. The nucleation and growth of these cracks ultimately lead to the alloy’s failure. Thus, pre-straining leads to the generation of additional micro-cracks, promotes the propagation of fatigue cracks, and results in reduced fatigue performance. Similar results have been observed in other studies [39,40].

However, the observation that the FCG rate of the pre-rolled samples was lower than that of the non-deformed sample contrasts with the previous analysis. This discrepancy arises from the fact that pre-rolling generates compressive residual stress, which can effectively enhance fatigue strength and FCG resistance [41]. As depicted in the results of FCG shown in Figure 2, it is clear that only mild pre-rolling is beneficial in reducing the FCG rate, while extensive pre-rolling results in a reduction in FCG resistance. This suggests that the effect of compressive residual stress on FCG resistance opposes the influence of work hardening. Notably, although the presence of compressive stress remains evident after 12% pre-rolling, the substantial deformation caused by the 12% pre-rolling permeates the entire sheet in the thickness direction (as shown in Figure 5). This results in a significant work hardening and a subsequent reduction in fatigue damage resistance.

In the case of the 9% pre-rolled sample, we achieved an optimal balance between compressive residual stress and work hardening, which leads to remarkable fatigue damage resistance. This excellent fatigue damage resistance can be ascribed to the non-uniform deformation introduced by pre-rolling. Specifically, after the 9% pre-rolling process, the majority of deformation was confined to the surface layer, with no modifications in the central layer. Consequently, the 9% pre-rolling yielded a compressive stress gradient throughout the thickness of the alloy, as evidenced in Figure 5. This non-uniform deformation also resulted in the uneven distribution of the T_1_ phase. As observed in Figure 6 and Table 2, the density of the T_1_ phase in the surface layer exceeded that in the central layer. Thus, it is evident that the combination of compressive stress distribution and the T_1_ phase created a unique hard surface and ductile center microstructure, resembling the effect of shot peening [42,43]. Undoubtedly, this distinctive microstructure resulting from the 9% pre-rolling process contributed to the exceptional fatigue damage resistance of the Al-Cu-Li alloy.

## 5. Conclusions

In this study, we examined the influence of pre-rolling at varying degrees (ranging from 0% to 12%) on the microstructure and fatigue crack propagation resistance of a third-generation Al-Li alloy. The investigation revealed a notable increase in tensile and yield strength as the degree of pre-rolling increased, while elongation progressively decreased. The alloy subjected to 9% pre-rolling exhibited the most favorable fatigue crack propagation rates due to a balanced effect of compressive residual stress and work hardening. Most of the deformation produced by this small pre-rolling were confined to the surface layer and did not extend to the central layer. This non-uniform deformation induced a compressive stress gradient in the thickness direction and led to an inhomogeneous distribution of T_1_ phase, resembling the structure generated by shot peening. These findings pave the way for future research aimed at optimizing alloy properties for diverse engineering and aerospace applications.

## Figures and Tables

**Table 1:** Room temperature mechanical properties of the Al-Cu-Li alloy in various pre-rolling conditions.

Pre-Deformation Conditions	Tensile Strength σ_b_ (MPa)	Yield Strength σ_0.2_ (MPa)	Elongation δ (%)
Without pre-rolling	475	393	15.1
3% pre-rolling	515	483	11.0
6% pre-rolling	523	495	9.87
9% pre-rolling	533	511	7.67
12% pre-rolling	546	525	6.53

**Table 2:** The average length of T_1_ phase in various pre-deformation conditions of Al-Cu-Li alloy.

Pre-Deformation Condition	Without Pre-Rolling	3% Pre-Rolling	6% Pre-Rolling	9% Pre-Rolling	12% Pre-Rolling
Average length of surface layer T_1_ phase (nm)	90	55	55	53	39
Average length of center layer T_1_ phase (nm)	145	84	62	61	39

## Data Availability

Data presented in this article are available at request from the corresponding author.
